# [Retracted] Tunicamycin inhibits progression of glioma cells through downregulation of the MEG-3-regulated wnt/β-catenin signaling pathway

**DOI:** 10.3892/ol.2025.15370

**Published:** 2025-11-03

**Authors:** Xin Li, Lei Xue, Qin Peng

Oncol Lett 15: 8470–8476, 2018; DOI: 10.3892/ol.2018.8416

Following the publication of the above paper, a concerned reader drew to the Editor's attention that the β-actin control blots shown for Figs. 4B and 5B held several protein bands in common, where different experimental conditions were described; moreover, the Lamin B data in Fig. 5A had apparently already appeared in a figure in a paper published in the journal *Oncotarget* that was written by different authors at different research institutes. Upon performing an independent analysis of the data in the Editorial Office, it came to light that the migration and invasion assay data shown in Fig. 2A-D and the immunofluorescence assay data in Fig. 4E and F were also strikingly similar to data that had already appeared in previously published papers in different journals written by different authors at different research institutes.

Given that the abovementioned data had already been published previously in a range of different journals, the Editor of *Oncology Letters* has decided that this article should be retracted from the Journal. The authors were asked for an explanation to account for these concerns, but the Editorial Office did not receive a reply. The Editor apologizes to the readership for any inconvenience caused.

